# Editorial: Autoimmune blistering diseases, volume II

**DOI:** 10.3389/fimmu.2023.1175962

**Published:** 2023-03-16

**Authors:** Xiaoguang Li, Giovanni Di Zenzo, Enno Schmidt, Pascal Joly, Takashi Hashimoto

**Affiliations:** ^1^ Department of Laboratory Medicine, Chronic Disease Research Center, Medical College, Dalian University, Dalian, China; ^2^ Laboratory of Molecular and Cell Biology, Istituto Dermopatico dell’Immacolata, IDI-IRCCS, Rome, Italy; ^3^ Lübeck Institute of Experimental Dermatology, University of Lübeck, Lübeck, Germany; ^4^ Department of Dermatology, University of Lübeck, Lübeck, Germany; ^5^ Department of Dermatology, Normandie University, UNIROUEN, Inserm U1234, CHU Rouen, Rouen, France; ^6^ Department of Dermatology, Osaka Metropolitan University Graduate School of Medicine, Osaka, Japan

**Keywords:** autoimmune blistering disease, pemphigus, pemphigoid, diagnosis, pathogenesis, treatment

Autoimmune blistering diseases (AIBDs) are intractable and potentially fatal autoimmune diseases, characterized clinically by cutaneous and/or mucosal blistering lesions. AIBDs are classified into two major groups, pemphigus and pemphigoid diseases, which show autoantibodies against keratinocyte cell surface proteins and epidermal/epithelial basal membrane zone (BMZ) proteins, respectively. Although major AIBD autoantigens have been identified and novel methods for detection of various autoantibodies/autoantigens have been developed, the final diagnoses cannot be made in a considerable number of AIBD cases, mainly because there are still some unidentified autoantigens. Various *in vitro* and *in vivo* experimental models for AIBDs have been established, shedding light on the pathogenic mechanisms. Key inflammatory pathways of most AIBDs are still incompletely understood, and safe and effective treatments remain to be established.

In the present Research Topic, 37 articles regarding diagnostic developments, pathogenic studies, evaluation of current treatment options, and new treatment protocols for AIBDs were collected.

For the convenience of the readers, we classified the articles into the 5 categories with the following subheadings.

(1) Diagnostic developments - 8 articles
(2) Studies on the pathogenesis - 16 articles
(3) Advances in treatment options – 4 articles
(4) Comprehensive summaries of large cohorts of specific AIBD subtypes
- 4 articles
(5) Reviews and opinions on AIBDs - 5 articles

## 1 Diagnostic developments

Kuang et al. presented a unique mucous membrane pemphigoid (MMP) case with only oral mucosal lesions, which revealed none of the known MMP autoantigens. However, this case showed autoantibodies against laminin γ1 and serum levels of anti-laminin γ1 IgA correlated with the disease activity. Therefore, laminin γ1 was considered as a novel MMP autoantigen, and the name of anti-laminin γ1 MMP was proposed. Another important finding in this patient was that autoantibodies against laminin α5 were also detected for the first time, although their pathogenic significance is currently unknown.

Qian et al. established novel ELISA assays with high sensitivities using peptides of desmoglein 1 (Dsg1), Dsg3 and BP180 as substrates, which were useful to identify the epitopes on the autoantigens. In this article, by combination with conventional ELISAs and immunoblotting methods, these novel ELISA assays were useful to follow a complicated AIBD patient for one year. This patient started as bullous pemphigoid (BP) and relapsed as concurrence of BP and mucosal-dominant-type pemphigus vulgaris (PV).

Recently, two indirect immunofluorescence tests have been developed, based on the expression of recombinant laminin 332 on the cell surface of HEK293 cells (biochip mosaic assay) and the migration trails of cultured keratinocytes rich in laminin 332 (keratinocyte footprint assay), respectively. The former test is highly standardized and widely available. Goletz et al. compared these two assays using sera of 54 anti-laminin 332 MMP patients and confirmed that both assays are highly sensitive and specific for diagnosis of anti-laminin 332 MMP. Testing for anti-laminin 332 serum IgG is of clinical importance, since a malignancy can be found in about a quarter of MMP patients with these autoantibodies.

Immune checkpoint inhibitors activate T-cell-mediated anti-tumor responses, whereas 1% of the treated patients developed immune checkpoint inhibitor-induced BP. Schauer et al. collected eight patients, who developed BP during or shortly after immune checkpoint inhibitor treatment. Analyses suggested that the immune checkpoint inhibitor-treated patients, who show abrupt onset of exanthematous or bullous eruptions, should be promptly screened by BP-specific diagnostic tests. Topical corticosteroids were sufficient in mild cases, but additional immunosuppressive agents were frequently required, while rituximab appeared as a good treatment option for severe cases.

Maglie et al. reported a rare female BP patient with concomitant localized scleroderma and cutaneous and genital lichen sclerosus. Similar cases in literature were reviewed, and possible immunological mechanisms were discussed. This case suggested that different diseases, i.e., BP and sclerosing dermatoses, might be caused by an immune activation against the same autoantigen.

Di Zenzo et al. reported a recessive dystrophic epidermolysis bullosa (DEB) patient with compound heterozygous null and missense COL7A1mutations, who experienced a rapid disease worsening accompanied by the onset of generalized severe itch. Positive tissue-bound and circulating IgG autoantibodies against BP180 led to the diagnosis of BP. The authors concluded that DEB patients presenting with rapid disease progression should be investigated for the development of a concomitant AIBD.

Schauer et al. reported 2 severe and recalcitrant female cases of mechanobullous-type epidermolysis bullosa acquisita (EBA). However, both patients also showed heterozygous recessive COL7A1 variants in a monoallelic state. Furthermore, type VII collagen (COL7) in cultured keratinocytes of these cases was significantly more vulnerable to proteolytic degradation than control keratinocytes, suggesting that the heterozygous pathogenic variants were sufficient to destabilize the molecule.

Lehr et al. screened anti-BP180 autoantibodies in 258 hereditary epidermolysis bullosa patients including 19 epidermolysis bullosa simplex, 8 junctional epidermolysis bullosa and 231 DEB patients. They found that 22% of the patients were positive for anti-BP180 autoantibodies, with higher positive rate (52.8%) in severe DEB. Among these patients, 6 (2.33%) patients showed clinical features of AIBD with positive results in indirect immunofluorescence. Combined analyses of the 6 cases and 4 similar cases in the literature suggested that hereditary epidermolysis bullosa patients have a predisposition for the development of AIBD, and therefore may be evaluated for coexistent AIBD.

## 2 Studies on the pathogenesis

Although MMP mucosal lesions, except for the lesions of the oral cavity, frequently lead to scarring, the mechanisms of scar development are largely unknown. Patzelt et al. found increased collagen fibril density in skin and conjunctival lesions in an antibody-transfer mouse model of anti-laminin 332 MMP, implying the changes in the collagenous matrix and cross-linking patterns involved in the mechanism of scar development in IgG-mediated MMP. However, further anti-fibrotic therapy using disulfiram, an inhibitor of the aldehyde dehydrogenase, failed to reduce the fibrosis or alleviate the disease activity on oral and ocular lesions of MMP mice, indicating that further studies with various anti-fibrotic treatments are required to clarify the mechanisms.

Bao et al. investigated the potential contribution of anti-laminin 332 autoantibodies to the expression of inflammatory factors in keratinocyte by RNA-seq of primary human keratinocytes stimulated by IgG anti-laminin 332 autoantibodies purified from patients with anti-laminin 332 MMP. Gene expressions of numerous cytokines and chemokines were upregulated. Further experiments using IgG autoantibodies against specific subunits of laminin 332 revealed upregulations of IL-1α and IL-6 at the protein level, most notably in keratinocytes treated with anti-laminin β3 antibodies.

Wang et al. performed an observational case-control study to explore the gene polymorphism of various cytokines and their clinical significance in 61 Chinese patients with BP. IL-13 showed significant differences in the nucleotide ratio, genotype, haploid frequency, and haplotype. Furthermore, IL-13 variants (rs20541, rs1800925) were related to gender, and the IL-13 genotype was significantly associated with recurrence. In addition, IL-13 concentrations in BP sera were significantly higher than those in healthy controls. These results indicated that IL-13 could be a potential target for therapy and marker for prognosis in BP.

To investigate the complement-related mechanism in BP, Emtenani et al. first performed a transcriptome analysis of perilesional and non-lesional skin biopsies of BP patients and identified upregulated expression of complement-associated genes, such as C5aR1 and C5aR2. In early skin lesions of BP patients, T cells and macrophages were found as the dominant cellular sources of C5aR1, while C5aR2 was mainly expressed on mast cells and eosinophils. Functional experiments indicated that C5a/C5aR1 axis was pivotal for attracting inflammatory cells to the skin, substantiating the involvement of C5a/C5aR1 axis in the pathogenesis of BP.

Wen et al. studied the potential contribution of scratching in the pathogenesis of experimental EBA in mice. As early as 12 hours after injection of anti-COL7 IgG into the skin of mice, an increased frequency of scratching appeared. Application of local anesthetic, dyclonine hydrochloride, before injection of anti-COL7 IgG could reduce the scratching events and improved clinical disease manifestation. However, administration of dyclonine hydrochloride 24 hours after injection of anti-COL7 IgG only inhibited the scratching behavior, but not clinical disease development. Therefore, scratching behavior may contribute to the initiation phase of disease manifestation in experimental EBA.

Tukaj et al. studied the potential pathogenic role of anti-heat shock protein 70 (Hsp70) autoantibodies in EBA. Compared to healthy controls, EBA patients had significantly elevated levels of circulating anti-Hsp70 IgG autoantibodies, which were positively correlated with serum levels of pro-inflammatory interferon gamma. Elevated serum levels of anti-Hsp70 IgG autoantibodies were also found in the passive transfer mouse model of EBA. In addition, anti-Hsp70 IgG antibody treatment led to pronounced dermal neutrophil infiltration and increased NF-κB activity in this model.

Ghorbanalipoor et al. employed three different mouse models of pemphigoid diseases, including antibody transfer-induced MMP, antibody transfer-induced EBA and immunization-induced EBA, to analyze the kinase activity. They found that PI3Kdelta was within the kinome activation network in antibody transfer-induced MMP and immunization-induced EBA, but not in antibody transfer-induced EBA. Parsaclisib, a selective inhibitor to PI3Kdelta, showed therapeutic effects in both antibody transfer-induced MMP and immunization-induced EBA, but not in autoantibody-induced EBA. These data indicated that cutaneous kinase activity of inflamed skin may correlate with treatment outcomes following PI3Kdelta inhibition in experimental mouse models of pemphigoid diseases.

IgG levels are maintained by IgG-recycling neonatal Fc-receptor (FcRn). To assess the impact of FcRn antagonism by efgartigimod on pemphigus-related immunological parameters, Maho-Vaillant et al. examined pemphigus patients during and after treatment with efgartigimod in combination with prednisolone. The treatment resulted in reduction of both total IgG and autoreactive IgG antibody levels. In several patients, autoreactive antibody levels remained low after stopping the efgartigimod treatment. Antigen-specific analyses revealed a loss of Dsg-specific B cells in several patients responding to efgartigimod, in line with prolonged reduction of pathogenic IgG levels. Therefore, efgartigimod treatment improved the pemphigus lesions *via* an immunomodulatory effect by the blockade of IgG recycling.

To study the pathogenesis of pemphigus foliaceus (PF), Hiermaier et al. analyzed the fate of membrane-bound Dsg1 in HaCaT cells, which were treated with IgG of PF patients. PF-IgG-induced loss of intercellular adhesion could be ameliorated by suppression of Ca^2+^ and ERK1/2 signaling. PF-IgG reduced the extra-desmosomal Dsg1, and remained Dsg1 was still located inside desmosomes. The intra-membrane mobility and localization of Dsg1 induced by PF-IgG was also reverted by suppression of Ca^2+^ signaling. Therefore, IgG anti-Dsg1 autoantibodies may contribute to the pathogenesis of PF by redistribution predominantly of membrane-bound Dsg1 at extra-desmosomal sites in a Ca^2+^ signaling dependent manner.

Schmitt et al. investigated the number of desmosomes and Dsg1 and Dsg3 composition in PV skin using super-resolution microscopy, with healthy skin as control. The number of desmosomes in patient skin was reduced significantly in the keratinocytes at basal and spinous layers, where only few split desmosomes were found. Desmosomes and Dsg1 and Dsg3 in extra-desmosomal sites were depleted predominantly in lower epidermis in PV. These results support the hypothesis that pemphigus is a desmosome assembly disease and may help to explain histopathologic differences among distinct pemphigus types.

Kugelmann et al. studied the role of sheddases, ADAM10 and ADAM17, both on the keratinocyte adhesion and on the pathogenesis of PV. In primary human keratinocytes, ADAM10 inhibition enhanced cell adhesion and shifted keratinocyte adhesion towards a hyperadhesive state, while ADAM10 did not modulate the protein levels of Dsg1 and Dsg3. In keratinocytes treated with IgG from mucocutaneous-type PV patients, ADAM10 inhibition reduced the loss of cell adhesion and fragmentation of Dsg1 and Dsg3. In keratinocytes treated with IgG from two mucosal-dominant-type PV and a monoclonal antibody against Dsg3, ADAM10 inhibition decreased fragmentation of cells in dissociation assay. However, these protective effects of ADAM10 inhibition were not observed when cells were treated with another PV-IgG containing more anti-Dsg1 autoantibodies.

In the studies of the potential contribution of UVA in the pathogenesis of PV by Eichkorn et al., they first showed that UVA induced the secretion of innate cytokines, including IL-1α, IL-1β, IL-6 and IL-8, in spontaneously immortalized keratinocyte cell line HaCaT. However, stimulation with PV-IgG alone did not result in a significant increase in cytokine release. Next, they showed that UVA enhanced PV-IgG induced acantholysis in a caspase dependent manner. Therefore, UVA is considered as a caspase-dependent exogenous cofactor for acantholysis, and local innate immune responses may contribute to overt clinical blister formation induced by autoantibodies of PV.

Drenovska et al. investigated the association of specific HLA alleles and haplotypes in 56 PV patients in the Bulgarian population, using 204 healthy individuals as control. HLA allele and haplotype distribution among Bulgarian patients with PV is similar to the already established results, i.e., predisposing associations with DRB1*14, DRB1*04:02, B*38, B*55, whereas DRB1*03:01 alleles and the corresponding haplotypes were significantly decreased in PV patients from the Bulgarian population. In addition, frequencies of PV-specific alleles HLA-A*01 and DRB1*11 were decreased in Bulgarian PV patients.

Baker et al. assessed correlations between factors including HLA genotype, ethnicity, autoantibody levels, and lesion distribution in a cohort of 293 PV patients. Patients typing as HLA DRB1*0402 had higher levels of anti-Dsg3 antibodies, while patients typing as DQB1*0503 had higher levels of anti-Dsg1 antibodies. The authors also identified an HLA association of DRB1*0804 in PV patients of African descent. Patients that carried neither DRB1*0402, nor DQB1*0503 or DRB1*0804 had the lowest levels of anti-Dsg3 antibodies and the highest rate of solely cutaneous disease compared to carriers of these alleles. These results indicated that differences of HLA expression and ethnicities play a large role on antibody selection and disease phenotype in PV.

Golinski et al. investigated the distribution and *in vitro* pathogenicity of anti-Dsg3 IgG subclasses during the disease course of PV. Percentages of patient sera, which contained 2 or more subclasses of anti-Dsg3 antibodies were 63% at baseline, 36% at complete remission stage, and 75% at persistent disease activity stage. The presence of 3 or more subclasses was a predictive factor for relapse, particularly when IgG3 antibodies were included. Both IgG4 and IgG3 subclasses of anti-Dsg3 antibodies had potentially pathogenic effects, particularly the higher levels of IgG4 antibodies. These results suggest that the serum levels and numbers of subclasses of IgG anti-Dsg3 antibodies predict the pathogenic effect of PV and the occurrence of relapses.

Wang et al. demonstrated the pathogenic roles of autoantibodies against the C-terminus of desmoplakin in paraneoplastic pemphigus (PNP) by using *in vitro* dispase-dependent keratinocyte dissociation assay and passive transfer neonatal mouse model, using IgG autoantibodies against C-terminus of desmoplakin purified from patient sera. These results further emphasize the importance of anti-desmoplakin autoantibodies in the pathogenesis of PNP.

## 3 Advances in treatment options

Rituximab efficacy has been demonstrated in small series of severe MMP cases refractory to conventional immunosuppressants. Bohelay et al. performed a retrospective single-center study on rituximab efficacy in 109 severe and/or refractory MMP patients with a median follow-up period of 51.4 months. The median periods to disease control and to complete remission were 7.1 months and 12.2 months, respectively. One year after rituximab weaning, complete remission could be obtained in two thirds of patients. Relapse occurred in about one third, and complete remission could be achieved again in most of these patients. In addition, 5 patients were non-responders to rituximab. This large cohort study with long term follow-up further confirmed both the efficacy and the limitations of rituximab in severe and/or refractory MMP.

Mise et al. reported on a single-center retrospective study about rituximab efficiency in pemphigus patients in Croatia. In total 19 patients with the mean follow-up time of 24.1 months were included. Thirteen out of 19 patients achieved complete remission during the study time. One patient developed a treatment-related-adverse event of infectious etiology (cellulitis). Rituximab was an effective treatment for pemphigus patients in Croatia with benefits of reductions of corticosteroid doses and steroid-related side effects, although repeated treatment cycles were often needed to achieve complete remission because of higher relapse rates. Optimized protocol of the rituximab treatment and identification of predictive markers for relapse may improve the management of pemphigus patients.

Zhang et al. studied the efficacy of dupilumab on moderate-to-severe BP patients by comparison of two groups, dupilumab group (8 patients) and conventional group (16 patients). No adverse event related to dupilumab was recorded. Compared to the conventional group, patients in the dupilumab group showed a shorter time for cessation of new blister formation, shorter time for the reduction of the systemic corticosteroids to minimal dose, and lower total amount of methylprednisolone and azathioprine. These results indicated that dupilumab combined with methylprednisolone/azathioprine might be superior to methylprednisolone/azathioprine alone in control of disease and tapering of corticosteroids for moderate-to-severe BP.

Alexandre et al. conducted a retrospective study to evaluate the effectiveness of omalizumab, a humanized monoclonal anti-IgE antibody, in 13 first-line therapy-resistant patients with BP or MMP with median total follow-up time of 30 months. This case series demonstrated that omalizumab is an effective biologic therapy for refractory BP and MMP, permitting rapid disease control and reduction of concomitant therapeutics, although 2 (15%) patients showed therapeutic failure.

## 4 Comprehensive summaries of large cohorts of specific AIBD subtypes

Qian et al. summarized and analyzed the clinical, pathological and immunological features of 133 anti-laminin 332-type MMP cases. Clinically, 89% and 43% patients had oral and ocular mucosal lesions, respectively, 71% had cutaneous lesions, and 17% had associated malignancies. Patient sera most frequently reacted with the laminin γ2 subunit (58%). In addition, one third of patients additionally reacted with non-laminin 332 autoantigens, which may contribute to the complexity in anti-laminin 332-type MMP.

Quintarelli et al. summarized clinical features, survivals, comorbidities, and treatment regimens in 149 pemphigus patients in Tuscany, Italy, for the recent 12 years. These patients included 108 PV, 35 PF, 3 PNP and 3 IgA pemphigus patients. Pemphigus patients showed a high incidence of serious comorbid diseases, including cerebro accidents, cardiovascular accidents and malignancies, highlighting the importance of a multidisciplinary approach for a proper management of pemphigus patients.

Anti-desmocollin (Dsc) antibodies were considered to be associated with several particular types of pemphigus, but have been rarely described in previous studies for various types of pemphigus. Bosch-Amate et al. conducted a systematic review by summarizing clinicopathological and immunological features and outcome in anti-Dsc autoantibodies-positive pemphigus patients. They collected 93 cases, including 38 (41%) cases with only anti-Dsc autoantibodies and 55 (59%) cases with anti-Dsc antibodies and other autoantibodies. In patients with only anti-Dsc autoantibodies, 17 cases had only IgG antibodies, 16 had only IgA antibodies, and 5 had both IgG and IgA antibodies. Both in patients with exclusive IgG anti-Dsc autoantibodies and in patients with both anti-Dsc antibodies and other autoantibodies, conventional pemphigus treatments and rituximab were good therapeutic options. For patients with IgA anti-Dsc autoantibodies, systemic corticosteroids followed by dapsone or retinoids were effective. In addition, cases with IgA or IgG/IgA antibodies were frequently associated with malignancies or other autoimmune diseases.

Pemphigoid nodularis is a rare form of pemphigoid with clinical features of prurigo nodularis and immunological features of BP. Szymanski et al. reported 5 female patients with confirmed pemphigoid nodularis with long time follow-up, and analyzed their clinical, immunological and therapeutic features. Although pemphigoid nodularis has been reported to be associated with drugs, these 5 patients could exclude drugs as provocative factors. Importantly, combination of topical clobetasol propionate on the entire body and antidepressants was effective in this study.

## 5 Reviews and opinions on AIBDs

Cole et al. reviewed complement-independent mechanisms involved in the pathogenesis of BP. Complement-independent pathways, such as macropinocytosis of IgG-BP180 complexes resulting in depletion of cellular BP180, may contribute to tissue damage and subepidermal blister formation in BP. These mechanisms may open new perspectives on novel targeted treatment modalities.

Xie et al. conducted a systematic review on coexistence of anti-p200 pemphigoid and psoriasis. A close association between psoriasis and anti-p200 pemphigoid has been demonstrated by previous studies. A total of 21 eligible studies comprising 26 anti-p200 pemphigoid patients with preceding psoriasis were involved. Compared with anti-p200 pemphigoid cases without psoriasis, more frequent involvement of the trunk and less mucosal involvement were illustrated in anti-p200 pemphigoid cases with psoriasis. Generally, monotherapy is sufficient for a complete remission for such patients. However, about a third of patients experienced at least one relapse, particularly those treated with prednisolone. Therefore, medication should be tapered carefully.

Egu et al. conducted a review on mechanisms of acantholysis in PV focusing on autoantibody profiles and their role in *in vitro* and *in vivo* models. The analyses indicated that investigations on desmosome composition might be helpful to promote understanding of the regulation of desmosome turnover, and that deciphering a signaling pathway may be druggable for PV as an additional line of therapy.

Li et al. conducted a mini review on the possible role of salivary proteins of insects in endemic PF in Brazil and Tunisia. In this review, not only the endemic PF features but also the corresponding autoantibody profiles were summarized. Pathways from insect bites to endemic PV were also described. These phenotypes of endemic PF observed in Brazil and Tunisia represent autoimmune diseases where the autoantibody response may be linked to an environmental etiology, i.e., salivary proteins from sand flies autochthonous to these countries.

Although vaccines are currently most important preventive measure against infectious diseases, vaccinations have also been accused of a potential risk on induction of autoimmune diseases. Russo et al. discussed the correlation of COVID-19 vaccines and ABIDs development firstly by citing a case of a 75-year-old male with type-II diabetes mellitus treated with gliptins, who developed BP 48 hours after receiving the first dose of the Comirnaty Pfizer-BioNTech vaccine. The authors suggest the role of vaccine as a trigger, but not a cause, of immune reaction in the setting of a gliptin-related BP. Benefits of vaccination against COVID-19 outweigh risks of development of AIBDs, and therefore, dermatologists should advise their patients to get vaccinated.

## 6 Concluding remarks

The 37 articles published in the present Research Topic for AIBDs help to improve the diagnosis by finding new autoantigens, to identify new disease phenotypes, to identify complicated concurrent diseases, to understand the pathogenesis in MMP, BP, EBA, PF, PV and PNP, and to know adequate and new treatments, including rituximab for MMP and pemphigus, as well as dupilumab and omalizumab for BP. The summaries of larger cohorts of anti-laminin 332-type MMP and various subtypes of pemphigus, as well as the reviews on unique perspectives for AIBDs, expand and deepen our understanding on AIBDs.

We hope that this Research Topic will be of interest to the scientific community, and advance the development of novel diagnostic methods and treatment of AIBDs.

## Author contributions

All authors listed have made a substantial, direct and intellectual contribution to the work, and approved it for publication.

